# Gastrointestinal bleeding in elderly patients with atrial fibrillation: prespecified All Nippon Atrial Fibrillation in the Elderly (ANAFIE) Registry subgroup analysis

**DOI:** 10.1038/s41598-024-59932-5

**Published:** 2024-04-27

**Authors:** Takatsugu Yamamoto, Yuji Mizokami, Takeshi Yamashita, Masaharu Akao, Hirotsugu Atarashi, Takanori Ikeda, Yukihiro Koretsune, Ken Okumura, Wataru Shimizu, Shinya Suzuki, Hiroyuki Tsutsui, Kazunori Toyoda, Atsushi Hirayama, Masahiro Yasaka, Takenori Yamaguchi, Satoshi Teramukai, Tetsuya Kimura, Yoshiyuki Morishima, Atsushi Takita, Hiroshi Inoue

**Affiliations:** 1 Department of Medicine, Teikyo University School of Medicine, 2-11-1 Kaga, Itabashi-ku, Tokyo, 173-8606 Japan; 2 Department of Gastroenterology, New Tokyo Hospital, Matsudo, Chiba Japan; 3 The Cardiovascular Institute, Minato-ku, Tokyo, Japan; 4 Department of Cardiology, National Hospital Organization Kyoto Medical Center, Fushimi-ku, Kyoto, Japan; 5 AOI Hachioji Hospital, Hachioji, Tokyo Japan; 6 Department of Cardiovascular Medicine, Toho University Faculty of Medicine, Ota-ku, Tokyo, Japan; 7 National Hospital Organization Osaka National Hospital, Chuo Ward, Osaka, Japan; 8 Division of Cardiology, Saiseikai Kumamoto Hospital Cardiovascular Center, Minami-ku, Kumamoto, Japan; 9 Department of Cardiovascular Medicine, Nippon Medical School, Tama Nagayama Hospital, Tama, Tokyo Japan; 10 Department of Cardiovascular Medicine, Faculty of Medical Sciences, Kyushu University, Higashi-ku, Fukuoka, Japan; 11 Department of Cerebrovascular Medicine, National Cerebral and Cardiovascular Center, Suita, Osaka Japan; 12 Department of Medicine, Osaka Fukujyuji Hospital, Neyagawa, Osaka Japan; 13 Department of Cerebrovascular Medicine, Fukuoka Neurosurgical Hospital, Minami-ku, Fukuoka, Japan; 14 Department of Biostatistics, Graduate School of Medical Science, Kyoto Prefectural University of Medicine, Kamigyo-ku, Kyoto, Japan; 15 Primary Medical Science Department, Daiichi Sankyo Co., Ltd., Chuo-ku, Tokyo, Japan; 16 Data Intelligence Department, Daiichi Sankyo Co., Ltd., Shinagawa-ku, Tokyo, Japan; 17 Saiseikai Toyama Hospital, Toyama, Toyama Japan

**Keywords:** Drug therapy, Outcomes research

## Abstract

Gastrointestinal (GI) bleeding control is critical in elderly patients with atrial fibrillation (AF) receiving oral anticoagulants (OAC). This subgroup analysis aimed to clarify the actual state and significance of GI bleeding in elderly non-valvular AF (NVAF) patients. We evaluated the incidence and risk factors of GI bleeding during the 2-year follow-up and examined the GI bleeding impact on mortality. Of the 32,275 patients in the ANAFIE Registry, 1139 patients (3.5%) experienced GI bleeding (incidence rate, 1.92 events per 100 person-years; mean follow-up, 1.88 years); 339 upper and 760 lower GI bleeding events occurred. GI bleeding risk factors included age ≥ 85 years, body mass index ≥ 25.0 kg/m^2^, prior major bleeding, hyperuricaemia, heart failure, P-glycoprotein inhibitor use, GI disease, and polypharmacy (≥ 5 drugs). No significant differences in GI bleeding risk were found between direct OAC (DOAC) vs warfarin users (adjusted hazard ratios [95% confidence interval], 1.01 [0.88–1.15]). The 1-year post-GI bleeding mortality rate was numerically higher in patients with upper (19.6%) than lower GI bleeding (8.9%). In elderly Japanese NVAF patients, this large-scale study found no significant difference in GI bleeding risk between DOAC vs. warfarin users or 1-year mortality after upper or lower GI bleeding.

## Introduction

Atrial fibrillation (AF) is a frequently occurring cardiac arrhythmia, which is increasing in incidence and prevalence in Japan because of the growing ageing population^1,2^. Both AF and older age are potent risk factors for ischaemic stroke associated with cardiogenic thromboembolism and the burden of disability^2,3^. Before 2010, vitamin K antagonists (VKA), such as warfarin, were the cornerstone for preventing stroke/systemic embolic events in patients with non-valvular AF (NVAF)^4,5^. However, warfarin is associated with an elevated risk of major bleeding (7.2 per 100 person-years), of which the most common type is gastrointestinal (GI) bleeding (nearly threefold increased risk of GI bleeding vs placebo)^6^.

Direct oral anticoagulants (DOAC) are increasingly being used as an alternative to VKA, given their better efficacy in reducing stroke and intracranial haemorrhage. Although some randomised controlled trials (RCTs) have reported a similar or higher risk of GI bleeding compared with warfarin^7–11^, whether the risk of GI bleeding with DOAC is comparable to VKA and the risk across types of DOAC remains debatable^12,13^. A recent prospective study in France reported that DOACs do not affect upper GI bleeding outcomes compared with VKA^12^. In a recent systematic review and network meta-analysis of 28 RCTs with 139,587 patients, a similar risk of GI bleeding between DOAC and warfarin was observed, and there was no risk difference between DOAC types^13^. Nonetheless, prospective data are scant on the incidence and risk factors of GI bleeding in elderly patients with NVAF treated with OACs in the real-world setting^14,15^. For patients with NVAF undergoing oral anticoagulant (OAC) therapy for stroke prevention, it is critical to control GI bleeding, particularly among elderly patients.

The All Nippon AF in the Elderly (ANAFIE) Registry was a prospective large-scale observational study aimed at elucidating the clinical status and prognosis of > 30,000 Japanese patients with NVAF aged ≥ 75 years^16–18^. This subgroup analysis of the ANAFIE Registry aimed to investigate the incidence and risk factors of GI bleeding, assess the impact of the types of OACs (DOAC vs warfarin), and evaluate the impact of GI bleeding on mortality in elderly Japanese patients with NVAF in a real-world setting.

## Results

### Patient disposition and characteristics

The characteristics of the patients in this subgroup analysis are presented in Table 1 and Supplementary Table S1. Of the overall population enrolled in the ANAFIE Registry (*N* = 32,275), 1139 patients (3.5%) experienced GI bleeding and a total of 1312 GI bleeding events occurred during the mean 1.88-year follow-up period. The incidence rate of GI bleeding was 1.92 per 100 person-years. Of the total 1139 patients with GI bleeding events, 339 (29.8%) cases were upper GI bleeding, 760 (66.7%) were lower GI bleeding, and 74 (6.5%) were unspecified. Overall, most patients (55.4%) were receiving a reduced dose of DOACs, followed by a standard dose (19.0%) and under-dose (18.1%), with only 4.0% and 3.5% of patients receiving an off-label or over-dose, respectively.

**Table 1:** Patient characteristics.

Characteristic	GI bleeding					
	Total *n* = 1139	Upper *n* = 339	Lower *n* = 760	Unspecified *n* = 74	None *n* = 31,136	*p*-value^†^
Age, years	82.1 ± 4.8	82.9 ± 4.8	81.7 ± 4.7	84.4 ± 5.3	81.4 ± 4.8	< 0.001
Body mass index, kg/m^2^	23.6 ± 3.9	23.5 ± 4.1	23.8 ± 3.9	22.7 ± 3.6	23.3 ± 3.6	0.012
History of major bleeding	115 (10.1)	33 (9.7)	80 (10.5)	6 (8.1)	1324 (4.3)	< 0.001
Use of anticoagulants	1080 (94.8)	322 (95.0)	720 (94.7)	71 (95.9)	28,750 (92.3)	0.002
DOAC	763 (70.6)	222 (68.9)	519 (72.1)	49 (69.0)	20,822 (72.4)	0.200
Standard dose	125 (19.0)	35 (17.9)	91 (20.5)	8 (19.0)	3701 (20.7)	0.419
Over-dose	23 (3.5)	5 (2.6)	16 (3.6)	3 (7.1)	675 (3.8)	
Reduced dose	364 (55.4)	120 (61.2)	233 (52.5)	21 (50.0)	9184 (51.5)	
Under-dose	119 (18.1)	28 (14.3)	89 (20.0)	7 (16.7)	3511 (19.7)	
Off-label dose	26 (4.0)	8 (4.1)	15 (3.4)	3 (7.1)	769 (4.3)	
Warfarin	316 (29.3)	99 (30.7)	201 (27.9)	22 (31.0)	7917 (27.5)	0.214
Concomitant use of antiplatelet agents	230 (21.1)	70 (21.3)	153 (21.1)	14 (20.3)	5474 (18.5)	0.032
Concomitant use of PPIs	448 (41.1)	127 (38.7)	303 (41.8)	37 (53.6)	11,379 (38.5)	0.085
Concomitant use of P-glycoprotein inhibitor	33 (3.0)	10 (3.0)	21 (2.9)	2 (2.9)	528 (1.8)	0.003
Comorbidities						
Hypertension	904 (79.4)	273 (80.5)	600 (78.9)	57 (77.0)	23,408 (75.2)	0.001
Diabetes mellitus	363 (31.9)	116 (34.2)	232 (30.5)	29 (39.2)	8370 (26.9)	< 0.001
Dyslipidaemia	500 (43.9)	149 (44.0)	337 (44.3)	30 (40.5)	13,228 (42.5)	0.343
Hyperuricaemia	339 (29.8)	108 (31.9)	220 (28.9)	25 (33.8)	6975 (22.4)	< 0.001
Chronic kidney disease	304 (26.7)	100 (29.5)	195 (25.7)	16 (21.6)	6401 (20.6)	< 0.001
Severe hepatic dysfunction	14 (1.2)	4 (1.2)	10 (1.3)	0 (0.0)	281 (0.9)	0.255
Myocardial infarction	92 (8.1)	27 (8.0)	60 (7.9)	10 (13.5)	1759 (5.6)	< 0.001
Heart failure	538 (47.2)	165 (48.7)	343 (45.1)	47 (63.5)	11,578 (37.2)	< 0.001
GI disease	453 (39.8)	128 (37.8)	316 (41.6)	31 (41.9)	9014 (29.0)	< 0.001
Number of medications	7.7 ± 3.5	7.9 ± 3.5	7.6 ± 3.6	8.4 ± 4.1	6.6 ± 3.1	< 0.001

Data are *n* (%) or mean ± standard deviation. ^†^Comparison of groups with (inclusive of patients with upper GI bleeding, lower GI bleeding, and unspecified site bleeding) and without GI bleeding.

GI, gastrointestinal.

In 1139 patients with GI bleeding, the mean age was 82.1 years, and 59.2% were men. A total of 39.8% of patients with GI bleeding also had GI disease complications. The average CHA_2_DS_2_-VASc and HAS-BLED scores were 4.8 and 2.0, respectively. In total, 94.8% of patients were prescribed anticoagulants (DOAC: 70.6%; warfarin: 29.3%); 41.1% of patients were also concomitantly prescribed proton-pump inhibitors (PPIs). Most patients with any type of GI bleeding were prescribed a reduced dose of DOACs (upper, 61.2%; lower, 52.5%; and unspecified, 50.0%), followed by a standard dose (upper, 17.9%; lower, 20.5%; and unspecified, 19.0%).

There were no significant differences in sex, systolic blood pressure, and AF type between patients with and without GI bleeding. However, compared with patients without GI bleeding, those with GI bleeding were significantly older, and had more frequent prior major bleeding, lower diastolic blood pressure, higher CHADS_2_, CHA_2_DS_2_-VASc, and HAS-BLED scores (*p* < 0.001), and a slightly higher proportion were receiving anticoagulants (*p* = 0.002). In addition to using more medications (*p* < 0.001), a slightly higher proportion of patients with GI bleeding were concomitantly using antiplatelet agents (*p* = 0.032).

### Study outcomes

**Figure 1:**
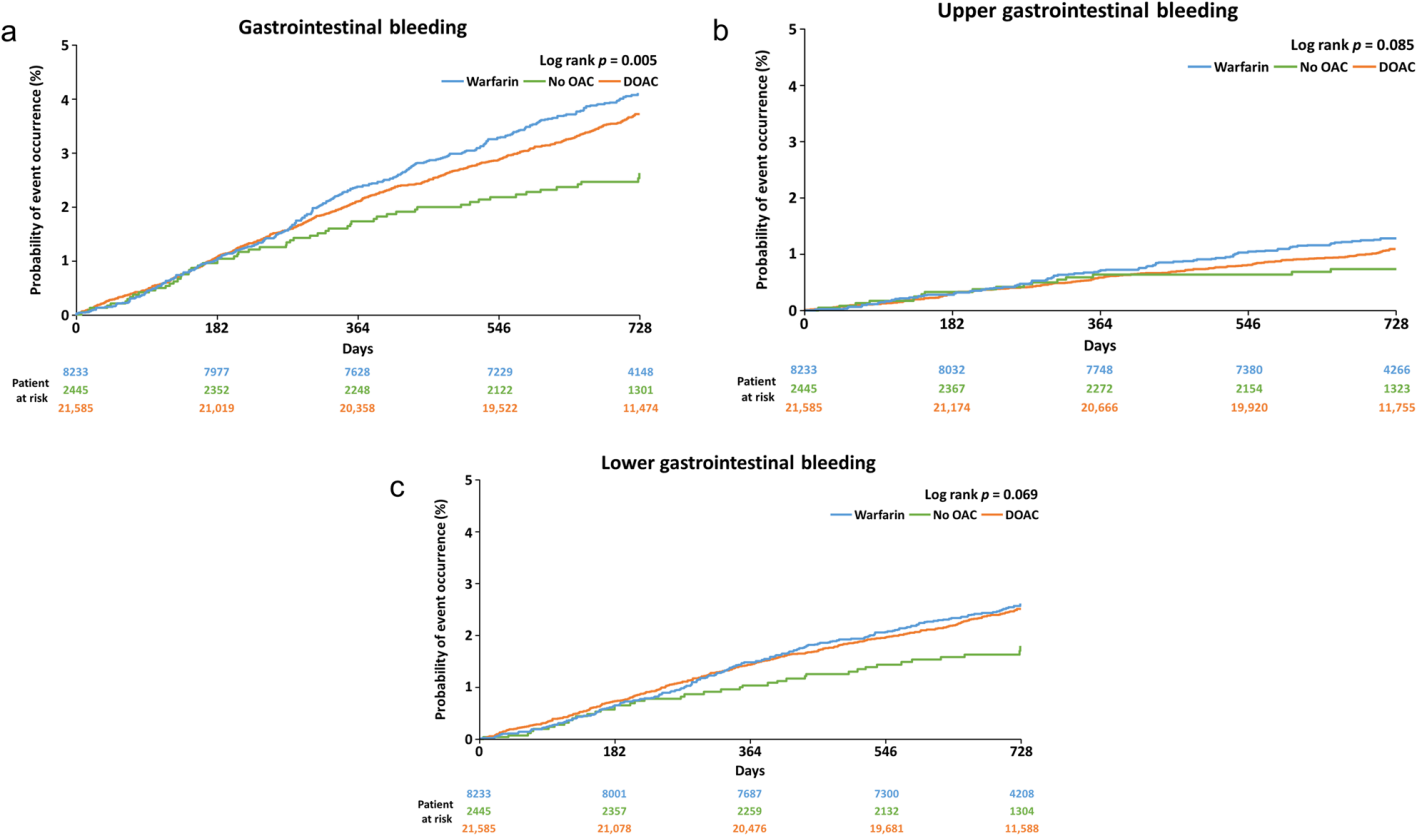
Kaplan–Meier event probability curves of (**a**) total, (**b**) upper, and (**c**) lower gastrointestinal bleeding by type of anticoagulant. DOAC, direct oral anticoagulant.

Table 2 shows the incidence of GI bleeding. Of the total of 1312 GI bleeding events in 1139 patients, the number of events of lower GI bleeding (*n* = 865, 65.9%) was numerically higher than that of upper GI bleeding (*n* = 370, 28.2%). The bleeding site was unknown for 77 (5.9%) events. Of the 1312 GI bleeding events, 21.1% (*n* = 277) were major bleeding, and 31.3% (*n* = 411) were minor bleeding per the ISTH definition. When categorised by bleeding site, major bleeding was numerically more frequent in the upper GI tract (upper: *n* = 132, 35.7% and lower: *n* = 123, 14.2%). Minor bleeding was numerically more common in the lower GI tract (*n* = 335, 38.7%) than in the upper GI tract (*n* = 54, 14.6%). Among patients taking warfarin or DOAC and those not taking OACs (no-OACs), a significant difference was observed in overall bleeding events (Fig. 1a), but no significant difference was observed when bleeding events were classified by site as upper GI bleeding (Fig. 1b) or lower GI bleeding (Fig. 1c).

**Table 2:** Number of bleeding events and classification by bleeding site.

	Total GI bleeding	Bleeding site		
		Upper	Lower	Unspecified
Patients with GI bleeding, *n*	1139	339	760	74
GI bleeding events, *n*	1312	370	865	77
Classification of bleeding, *n (%)*				
Major bleeding	277 (21.1)	132 (35.7)	123 (14.2)	22 (28.6)
Clinically relevant non-major bleeding	624 (47.6)	184 (49.7)	407 (47.1)	33 (42.9)
Minor bleeding	411 (31.3)	54 (14.6)	335 (38.7)	22 (28.6)

GI, gastrointestinal.

**Figure 2:**
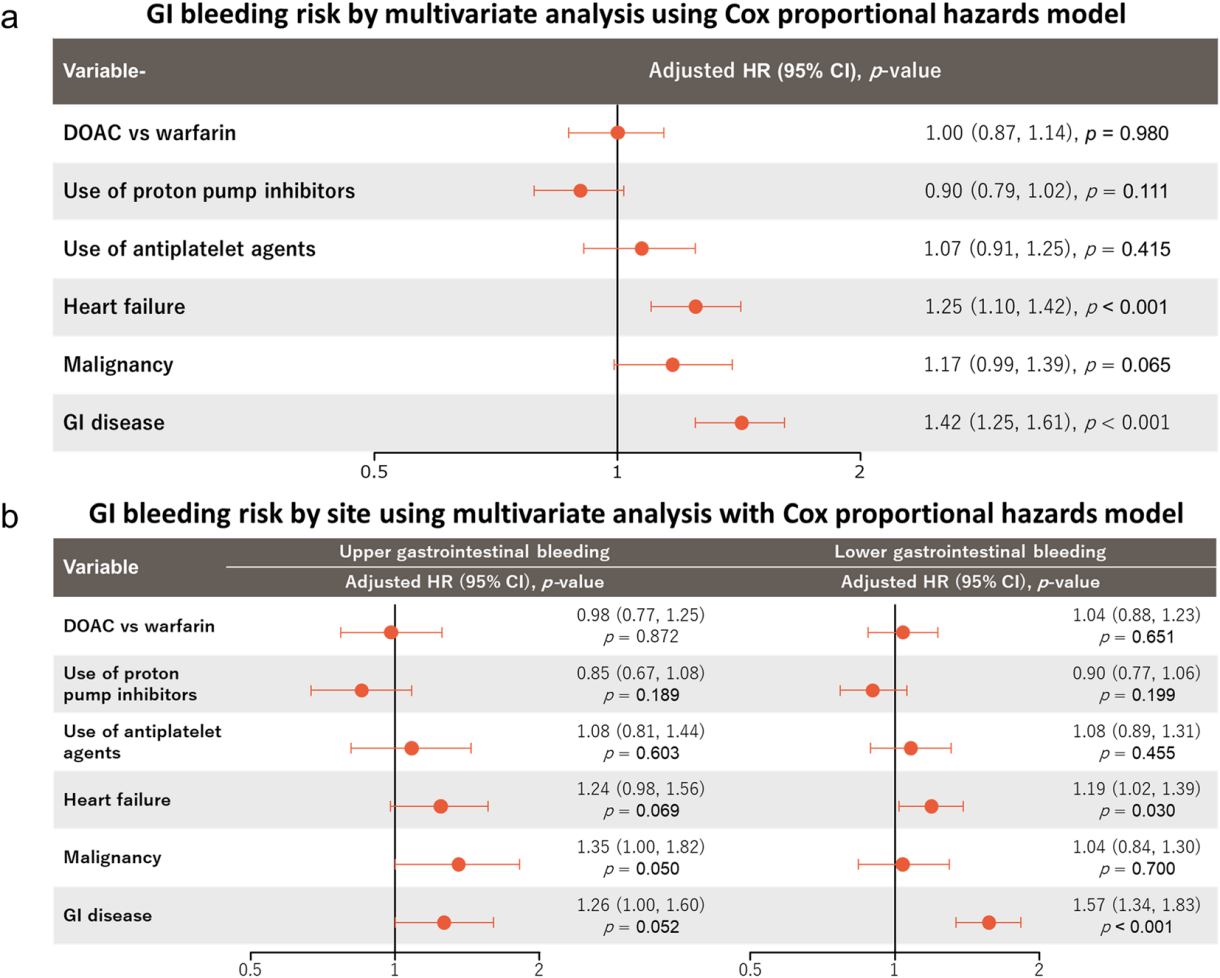
Forest plot of multivariate analysis in the Cox proportional hazards model for gastrointestinal bleeding by site. Adjusting factors: age, sex, body mass index, history of bleeding, type of AF, systolic blood pressure, severe hepatic disease, diabetes, hyperuricaemia, heart failure and/or reduced left ventricular ejection fraction, myocardial infarction, cerebrovascular disease, thromboembolic disease, active cancer, dementia, fall within 1 year, anticoagulants, history of catheter ablation, creatinine clearance, digestive diseases, polypharmacy, and use of antiarrhythmic drugs, antiplatelet agents, proton pump inhibitors, P-glycoprotein inhibitors, and anti-hyperlipidaemia drugs. CI: confidence interval, DOAC: direct oral anticoagulants, GI: gastrointestinal, HR: hazard ratio.

For the 1312 GI bleeding events, the HRs for total GI bleeding, upper GI bleeding, and lower GI bleeding are reported in Fig. 2 and Supplementary Table S2. No significant differences in the risk of GI bleeding were found between patients receiving DOAC and those receiving warfarin, whether it be upper GI bleeding (HR: 0.98, 95% CI: 0.77–1.25) or lower GI bleeding (1.04, 0.88–1.23). Similarly, excluding patients using DOAC off-label doses did not result in significant differences in the risk for either upper GI bleeding (1.00, 0.77–1.29) or lower GI bleeding (1.04, 0.87–1.24) (Supplementary Table S2). Furthermore, there was a trend toward a reduction of GI bleeding risk among patients using PPIs. Upper GI bleeding risk was higher in cancer patients (1.35, 1.00–1.82). The risk of lower GI bleeding was higher in patients with heart failure (1.19, 1.02–1.39) and GI disease (1.57, 1.34–1.83).

**Figure 3:**
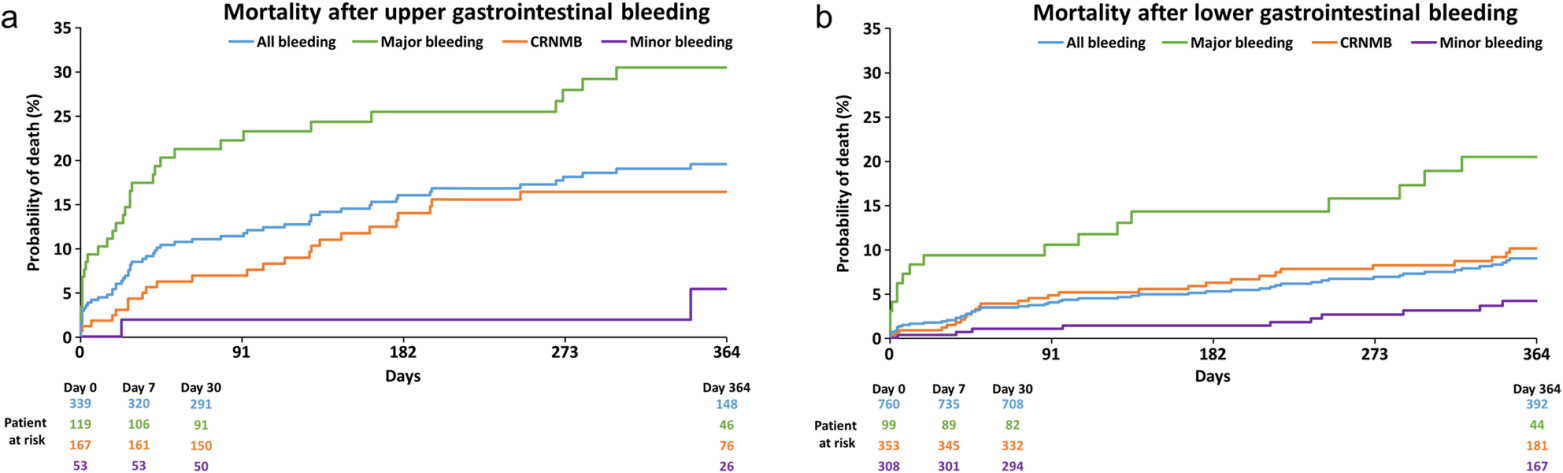
Kaplan–Meier for event probability of death 1 year after (**a**) upper gastrointestinal bleeding and (**b**) lower gastrointestinal bleeding. CRNMB, clinically relevant non-major bleeding.

Table 3 presents the risk factors associated with GI bleeding and with upper and lower GI bleeding specifically. In multivariate analysis, risk factors for GI bleeding included age ≥ 85 years (1.21, 1.06–1.39), body mass index (BMI) ≥ 25.0 kg/m^2^ (1.17, 1.01–1.35), history of major bleeding (2.22, 1.82–2.71), heart failure (1.24, 1.09–1.40), hyperuricaemia (1.18, 1.03–1.36), GI disease (1.41, 1.24–1.60), use of P-glycoprotein inhibitors (1.55, 1.09–2.20), and polypharmacy (use of 5–8 drugs: 1.23, 1.04–1.46; use of ≥ 9 drugs: 1.65, 1.35–2.01). Concomitant use of PPIs (0.88, 0.77–1.00) was a protective factor against GI bleeding. Risk factors for upper GI bleeding were age ≥ 85 years (1.67, 1.31–2.13), history of major bleeding (1.99, 1.38–2.88), and polypharmacy (≥ 9 drugs: 1.73, 1.21–2.47), and risk factors for lower GI bleeding were BMI ≥ 25.0 kg/m^2^ (1.28, 1.08–1.52), history of major bleeding (2.37, 1.87–3.01), heart failure (1.18, 1.01–1.37), GI disease (1.56, 1.34–1.82), and polypharmacy (5–8 drugs; 1.29, 1.05–1.59; ≥ 9 drugs: 1.61, 1.26–2.05). A protective factor against lower GI bleeding was no-OACs (0.70, 0.49–0.99) compared with warfarin. The mortality 1 year after the incidence of GI bleeding was numerically higher in patients with upper GI bleeding (19.6%, 95% CI: 15.4–24. 7) than in those with lower GI bleeding (8.9%, 6.9–11.5) (Fig. 3). The most common causes of death in those with overall GI bleeding and upper GI bleeding were the same, namely extracranial bleeding (overall: 24.2%; upper: 22.5%), malignancy (overall: 13.7%; upper: 22.5%), infection (overall: 13.7%; upper: 15.0%), and other causes (overall: 22.6%; upper: 17.5%), including undetermined, natural death from old age, and others. The most common causes of death for those with lower GI bleeding differed from those with upper GI bleeding; fewer deaths were due to extracranial bleeding (7.8%), and more deaths were due to other causes (25.5%), including undetermined causes and natural death (Table 4).

**Table 3:** Analysis of risk factors for GI bleeding by Cox proportional hazards model.

Factors	Total GI bleeding		Upper GI bleeding		Lower GI bleeding	
	HR (95% CI)	*p*-value	HR (95% CI)	*p*-value	HR (95% CI)	*p*-value
Female	0.92 (0.81, 1.04)	0.182	0.75 (0.59, 0.96)	0.020	0.99 (0.85, 1.16)	0.884
Age ≥ 85 years	1.21 (1.06, 1.39)	0.006	1.67 (1.31, 2.13)	< 0.001	1.00 (0.84, 1.19)	0.972
Body mass index ≥ 25.0 kg/m^2†^	1.17 (1.01, 1.35)	0.036	1.01 (0.77, 1.32)	0.968	1.28 (1.08, 1.52)	0.005
History of major bleeding	2.22 (1.82, 2.71)	< 0.001	1.99 (1.38, 2.88)	< 0.001	2.37 (1.87, 3.01)	< 0.001
Heart failure	1.24 (1.09, 1.40)	< 0.001	1.22 (0.96, 1.53)	0.100	1.18 (1.01, 1.37)	0.040
Hyperuricaemia	1.18 (1.03, 1.36)	0.017	1.22 (0.95, 1.57)	0.117	1.17 (0.99, 1.39)	0.068
Persistent AF^‡^	0.85 (0.71, 1.01)	0.067	0.89 (0.64, 1.23)	0.473	0.84 (0.67, 1.05)	0.120
Long-standing persistent + permanent AF^‡^	0.92 (0.81, 1.06)	0.254	0.96 (0.75, 1.23)	0.763	0.91 (0.77, 1.08)	0.282
Systolic blood pressure ≥ 140 mmHg	1.00 (0.85, 1.17)	0.989	0.90 (0.67, 1.22)	0.508	1.02 (0.84, 1.23)	0.841
Presence of GI disease	1.41 (1.24, 1.60)	< 0.001	1.25 (0.99, 1.58)	0.066	1.56 (1.34, 1.82)	< 0.001
No-OACs^§^	0.66 (0.50, 0.88)	0.005	0.64 (0.38, 1.09)	0.098	0.70 (0.49, 0.99)	0.043
Use of DOAC^§^	1.01 (0.88, 1.15)	0.941	0.99 (0.77, 1.26)	0.935	1.05 (0.88, 1.24)	0.607
Concomitant use of PPI	0.88 (0.77, 1.00)	0.044	0.81 (0.64, 1.03)	0.092	0.88 (0.75, 1.03)	0.122
Concomitant use of P-gp inhibitors	1.55 (1.09, 2.20)	0.014	1.59 (0.84, 2.99)	0.155	1.46 (0.94, 2.26)	0.092
Polypharmacy (use of 5–8 drugs)	1.23 (1.04, 1.46)	0.018	1.09 (0.79, 1.50)	0.600	1.29 (1.05, 1.59)	0.017
Polypharmacy (use of ≥ 9 drugs)	1.65 (1.35, 2.01)	< 0.001	1.73 (1.21, 2.47)	0.003	1.61 (1.26, 2.05)	< 0.001

^†^Versus body mass index > 18.5 kg/m^2^ and < 25.0 kg/m^2^.

^‡^Versus paroxysmal AF.

^§^Versus warfarin.

^¶^Versus polypharmacy (use of < 5 drugs).

AF, atrial fibrillation; CI, confidence interval; DOAC, direct oral anticoagulants; GI, gastrointestinal; HR, hazard ratio; OAC, oral anticoagulants; P-gp, P-glycoprotein; PPI, proton-pump inhibitors.

**Table 4:** Causes of death.

Cause of death	GI bleeding (*n* = 124)	Bleeding site	
		Upper (*n* = 40)	Lower (*n* = 51)
Cardiovascular death			
Fatal bleeding	32 (25.8)	9 (22.5)	5 (9.8)
Intracranial bleeding	2 (1.6)	0 (0.0)	1 (2.0)
Extracranial bleeding	30 (24.2)	9 (22.5)	4 (7.8)
Non-haemorrhagic cardiovascular death	23 (18.5)	8 (20.0)	13 (25.5)
Ischaemic stroke	4 (3.2)	1 (2.5)	0 (0.0)
Congestive heart failure or cardiogenic shock	9 (7.3)	3 (7.5)	8 (15.7)
Pulmonary embolism	2 (1.6)	1 (2.5)	0 (0.0)
Sudden or unwitnessed death	5 (4.0)	3 (7.5)	4 (7.8)
Other causes	3 (2.4)	0 (0.0)	1 (2.0)
Death caused by malignancy			
Malignancy	17 (13.7)	9 (22.5)	10 (19.6)
Other deaths	52 (41.9)	14 (35.0)	23 (45.1)
Infection	17 (13.7)	6 (15.0)	5 (9.8)
Renal disease	7 (5.6)	1 (2.5)	5 (9.8)
Other causes (i.e., undetermined, natural death, and others)	28 (22.6)	7 (17.5)	13 (25.5)

Data are *n* (%).

GI, gastrointestinal.

## Discussion

The main findings of this study were as follows. First, of the overall ANAFIE Registry population, 3.5% of elderly patients with NVAF experienced GI bleeding during a mean of 1.88 years of follow-up (1.92 events per 100 person-years). Of the 1312 GI bleeding events, 21.1% were major bleeding, 47.6% were CRNMB, and 31.3% were minor bleeding events. Additionally, there were 339 and 760 cases of upper and lower GI bleeding, respectively. Second, GI bleeding risk factors included age ≥ 85 years, body mass index ≥ 25.0 kg/m^2^, prior major bleeding, hyperuricaemia, heart failure, P-glycoprotein inhibitor use, GI disease, and polypharmacy (≥ 5 drugs). Third, no significant differences in the risk of GI bleeding were found between patients receiving DOAC vs those receiving warfarin. Finally, the mortality 1 year after the incidence of GI bleeding was numerically higher in patients with upper GI bleeding than in those with lower GI bleeding.

The overall incidence of GI bleeding was 3.5% in a mean of 1.88 years of follow-up in this study, which is higher than the previously reported incidence in Japan (2.1% during 39.3 months of follow-up)^19^ and overseas (1.8% for 360 days)^20^. However, the incidence rate of GI bleeding (1.92 events per 100 person-years) was similar to the previously reported rate (2.0% per year) in Japan^21^. Although previous reports from Japan^19^ showed a higher incidence of upper GI bleeding (44%) compared with lower GI bleeding (33%), the current results (i.e., more frequent lower GI bleeding) align with published studies conducted overseas^20^.

Published data from RCTs show that, compared with warfarin, the use of DOAC is associated with a similar or higher incidence of GI bleeding^11,22–24^. In contrast, the current study did not show any differences in GI bleeding between DOAC and warfarin, which is consistent with published data from Japan^19^. It is possible that the presence of cases with unknown sources of bleeding, which accounted for 5.9% of bleeding cases, may have influenced the findings. In this study, we observed a high rate of patients receiving PPIs during anticoagulation therapy for upper GI bleeding, which could have helped reduce bleeding. The study subjects were elderly patients, including possible cases in which malignant diseases caused GI bleeding. As some of these patients may bleed even without anticoagulation therapy, medication may have affected the results.

Antiplatelet agents are generally associated with an increased risk of bleeding, particularly GI bleeding^25^. However, there was no difference in the GI bleeding risk with versus without using concomitant antiplatelet agents in this study. A reasonable explanation for this result could be that the higher rate of PPI prescriptions among patients on antiplatelet agents^26^ may have masked the correlation between antiplatelet agents and GI bleeding^27^. Published studies have reported that concomitant PPI use reduced GI bleeding associated with OAC use^28^. In this study, the difference in concomitant PPI use was only 3% between patients with and without bleeding; thus, it is necessary to consider whether and the extent to which PPI use is associated with the risk of GI disease. Furthermore, although there was no difference in the GI bleeding risk with versus without the use of concomitant antiplatelet agents in this study, another study reported that concomitant use of antiplatelet agents and OACs is associated with an increased risk of major bleeding, including GI bleeding^29^. Thus, physicians should exercise caution when prescribing these therapeutic agents for concomitant use to minimise the risk of GI bleeding.

This ANAFIE Registry subgroup analysis identified several risk factors associated with GI bleeding in elderly Japanese patients with NVAF. These factors included age ≥ 85 years, BMI ≥ 25.0 kg/m^2^, history of major bleeding, heart failure, hyperuricaemia, GI disease, use of P-glycoprotein inhibitors, and polypharmacy (use of ≥ 5 drugs). A Japanese study revealed similar risk factors, including initiation of DOAC at ≥ 85 years of age, chronic kidney disease, low‑dose aspirin and nonsteroidal anti‑inflammatory drugs, and histories of GI bleeding and cancer^30^. Another Japanese study found overlapping factors such as age and impaired kidney function (creatinine ≥ 0.93 mg/dL), but also found that haemoglobin < 13.2 g/dL was also associated with GI bleeding risk^19^. In contrast, the risk factors identified in a study of patients prescribed DOAC conducted outside Japan were active cancer, abnormal renal function, bleeding predisposition, chronic obstructive pulmonary disease, and uncontrolled hypertension^31^. Reasons for differences in risk factors may be racial/ethnic differences among White and Asian patients with AF. A multicohort study of hospitalised AF patients showed that although patients of all races had an increased risk of intracranial haemorrhage with warfarin, non-White patients, particularly Asian patients, had a twofold to fourfold risk of warfarin-related intracranial haemorrhage^32^. Another more recent study reported that Asian patients, particularly Japanese patients with AF, had a significantly lower incidence of GI bleeding and ischaemic stroke when treated with the DOAC compared with warfarin^33^.

Of note, the risk factors for upper and lower GI bleeding differed in the present study. Risk factors for upper GI bleeding were age ≥ 85 years, history of major bleeding, and polypharmacy with ≥ 9 drugs. In contrast, risk factors for lower GI bleeding were BMI ≥ 25.0 kg/m^2^, history of major bleeding, heart failure, GI disease, and polypharmacy (5–8 drugs and ≥ 9 drugs). This finding is consistent with a study in Japan in which the characteristics of patients with upper and lower GI bleeding also differed^21^. Those with upper GI bleeding had a history of peptic ulcer, and those with lower GI bleeding used concomitant nonsteroidal anti-inflammatory drugs and antiplatelet agents^21^. The findings emphasise the implication of identifying risk factors for GI bleeding among elderly Japanese patients with NVAF, specifically for upper and lower GI bleeding. As most upper GI bleeding is related to gastric acid secretion, it can be prevented with gastric acid secretion inhibitors such as PPIs and potassium-competitive acid blockers. Conversely, effective preventive measures against lower GI bleeding have not been established. Furthermore, our results show that upper GI bleeding has a high risk of death. Based on these facts, we believe that it would be of great significance if it were possible to predict different risks for upper and lower GI bleeding. This knowledge is crucial for clinicians prescribing OACs to elderly Japanese patients with NVAF for GI bleeding prevention.

In the overall ANAFIE Registry population, cardiovascular disease (32.4%), infection (17.1%), and malignancy (16.1%) were the most common causes of death^34^. In the current study, most deaths were due to extracranial bleeding, cancer, or infection. Similarly, a published article on GI bleeding from OAC therapy among Japanese patients with AF reported that the leading causes of death were GI bleeding, pneumonia, cancer, heart failure, and sudden death^19^. The most common causes of death in elderly Japanese patients with NVAF with upper GI bleeding (i.e., extracranial bleeding, cancer, and infection) differed from those with lower GI bleeding, who had fewer deaths due to extracranial bleeding and more deaths due to other causes (including undetermined causes and natural death); this finding has important implications for patient prognosis.

The limitations of the ANAFIE Registry have been previously described^18^. One limitation of this subgroup analysis is that there were no data on the type or dose of OAC used at the onset of GI bleeding. Furthermore, there was no evaluation of patients with continued OAC use after the onset of GI bleeding. Furthermore, no significant difference was observed in the group with BMI < 18 kg/m^2^, but there was a considerable amount of bleeding. Therefore, an unknown confounding factor, such as ethnicity, may not have been adjusted for. Finally, this subgroup analysis did not evaluate *H. pylori* infection, which is largely responsible for GI bleeding.

In conclusion, this large-scale study found the incidence and risk factors for GI bleeding in elderly Japanese patients with NVAF. Furthermore, in patients receiving DOAC vs warfarin, there was no significant differences in the risk of GI bleeding and upper or lower GI bleeding. One-year mortality after GI bleeding may increase in patients with upper vs lower GI bleeding. This subanalysis of the ANAFIE Registry will enable clinicians to understand better and manage the risk factors for GI bleeding associated with anticoagulant therapy in elderly patients with NVAF and manage the post-GI bleeding prognosis in these patients.

## Methods

### Study design and participants

The full study design of the ANAFIE registry^17^ has been previously published. The clinical study was registered in the UMIN Clinical Trial Registry (UMIN000024006).

Briefly, the ANAFIE Registry was a multicentre, prospective, observational study. Patients were followed up for 2 years, and treating physicians prescribed pharmacotherapy based on their judgement, current clinical practices, current Japanese treatment guidelines, and approved drug indications per the current package insert labelling in Japan. The study complied with the Declaration of Helsinki and local requirements for registries and ethical guidelines for clinical studies in Japan. Ethical approval by the relevant institutional review boards was obtained, as was written informed consent from each patient or a family member if the patient had a communication disorder, such as aphasia, or cognitive impairment.

The main inclusion criteria of the ANAFIE Registry were as follows: male or female patients aged ≥ 75 years at the time of informed consent with a definitive diagnosis of NVAF, who could attend hospital visits and provide informed consent in writing.

Patients who met the following criteria were excluded from participating in this study: those currently participating or were planning to participate in an interventional study; with a definitive diagnosis of mitral stenosis; those having an artificial heart valve (mechanical valve or tissue valve prostheses); who experienced any cardiovascular event (stroke, myocardial infarction, cardiac intervention for cardiac events other than myocardial infarction, or heart failure requiring hospitalisation) or any bleeding leading to hospitalisation within 1 month before enrolment; and who were diagnosed as having a life expectancy of < 1 year due to disease.

The current study is a prespecified subgroup analysis of the ANAFIE Registry that focused on patients who experienced GI bleeding during the 2-year follow-up.

### Study endpoints

The subgroup analysis endpoints were the incidence of GI bleeding and mortality up to 1 year after the incidence of GI bleeding. Diagnostic procedures for GI bleeding were not strictly standardized and were performed at the discretion of the investigator. In general, if symptoms of GI bleeding appeared, the patient was assessed by endoscopy, the bleeding was found by the originally planned test, or the procedure was selected by the treating physician based on the patient’s symptoms. All clinical outcome events were adjudicated by event evaluation committees, all of whom were blind to the anticoagulation treatment. Bleeding events were categorised according to the International Society on Thrombosis and Haemostasis (ISTH) definition^35^.

### Statistical analysis

Categorical variables were analysed using frequency tables, and summary statistics were calculated for continuous variables and included n, mean, and standard deviation. The t-test was used to compare continuous variables and the chi-square test for categorical variables.

The probability of event occurrence during the 2-year follow-up period was estimated using the Kaplan–Meier method, and the corresponding *p*-values were calculated using the log-rank test. Incidence rates per 100 person-years with 95% confidence intervals (CIs) were also estimated. Patient prognosis after the occurrence of a GI bleeding event was evaluated by the Kaplan–Meier method within 1 year of GI bleeding event onset until the time of death.

Hazard ratios were estimated using the Cox proportional hazards model adjusted by patient background characteristics. A *p*-value < 0.05 was considered statistically significant. In multivariate analysis, the type of anticoagulant was incorporated into the model to analyse risk factors for GI bleeding, which resulted in the exclusion of 12 patients with parenteral anticoagulant prescriptions from the analysis. The statistical software used for analysis was SAS version 9.4 (SAS Institute, Tokyo, Japan).

### Supplementary Information

Supplementary Tables.

## Data Availability

1. Will the individual deidentified participant data (including data dictionaries) be shared? → Yes. 2 What data in particular will be shared? → Individual participant data that underlie the results reported in this article, after deidentification (text, tables, figures, and appendices). 3 Will any additional, related documents be available? If so, what is it? (e.g., study protocol, statistical analysis plan, etc.) → Study Protocol. 4 When will the data become available and for how long? → Ending 36 months following article publication. 5 By what access criteria will the data be shared (including with whom)? → The access criteria for data sharing (including requests) will be decided by a committee led by Daiichi-Sankyo. 6 For what types of analyses, and by what mechanism will the data be available? → Any purpose: Proposals should be directed to yamt-tky@umin.ac.jp. To gain access, data requestors will need to sign a data access agreement.
