# Supplementary material for: Gastrointestinal bleeding in elderly patients with atrial fibrillation: prespecified All Nippon Atrial Fibrillation in the Elderly (ANAFIE) Registry subgroup analysis
Source: Sci Rep. 2024 Apr 27;14:9688. doi: 10.1038/s41598-024-59932-5 (PMC11055876; doi:10.1038/s41598-024-59932-5)
Supplement: Supplementary file 1 — Supplementary Tables. [file 41598_2024_59932_MOESM1_ESM.docx]

**Supplementary Information**

# Gastrointestinal bleeding in elderly patients with atrial fibrillation: Prespecified All Nippon Atrial Fibrillation in the Elderly (ANAFIE) Registry subgroup analysis

Takatsugu Yamamoto, Yuji Mizokami, Takeshi Yamashita, Masaharu Akao, Hirotsugu Atarashi, Takanori Ikeda, Yukihiro Koretsune, Ken Okumura, Wataru Shimizu, Shinya Suzuki, Hiroyuki Tsutsui, Kazunori Toyoda, Atsushi Hirayama, Masahiro Yasaka, Takenori Yamaguchi, Satoshi Teramukai, Tetsuya Kimura, Yoshiyuki Morishima, Atsushi Takita, Hiroshi Inoue

**Supplementary Table S1.** Additional patient characteristics.

| **Characteristic** | **GI bleeding** | | | | | |
| --- | --- | --- | --- | --- | --- | --- |
|  | **Total**  **n = 1,139** | **Upper**  **n = 339** | **Lower**  **n = 760** | **Unspecified**  **n = 74** | **None**  **n = 31,136** | ***p*-value^†^** |
| Male | 674 (59.2) | 213 (62.8) | 441 (58.0) | 36 (48.6) | 17,808 (57.2) | 0.185 |
| Age, years | 82.1 ± 4.8 | 82.9 ± 4.8 | 81.7 ± 4.7 | 84.4 ± 5.3 | 81.4 ± 4.8 | <0.001 |
| ≥75 to <80 years | 391 (34.3) | 94 (27.7) | 286 (37.6) | 16 (21.6) | 12,504 (40.2) |  |
| ≥80 to <85 years | 396 (34.8) | 116 (34.2) | 272 (35.8) | 22 (29.7) | 10,565 (33.9) |  |
| ≥85 to <90 years | 258 (22.7) | 93 (27.4) | 154 (20.3) | 21 (28.4) | 6037 (19.4) |  |
| ≥90 to <100 years | 94 (8.3) | 36 (10.6) | 48 (6.3) | 15 (20.1) | 2019 (6.4) |  |
| ≥100 years | 0 (0.0) | 0 (0.0) | 0 (0.0) | 0 (0.0) | 11 (0.0) |  |
| Systolic blood pressure, mmHg | 126.8 ± 17.0 | 126.1 ± 17.5 | 127.1 ± 16.6 | 127.1 ± 17.1 | 127.4 ± 17.0 | 0.290 |
| Diastolic blood pressure, mmHg | 68.9 ± 12.0 | 67.5 ± 11.4 | 69.7 ± 12.2 | 67.6 ± 12.3 | 70.7 ± 11.6 | <0.001 |
| CHADS_2_ score | 3.1 ± 1.3 | 3.2 ± 1.2 | 3.1 ± 1.3 | 3.4 ± 1.3 | 2.8 ± 1.2 | <0.001 |
| CHA_2_DS_2_-VASc score | 4.8 ± 1.5 | 4.9 ± 1.5 | 4.7 ± 1.5 | 5.2 ± 1.5 | 4.4 ± 1.4 | <0.001 |
| HAS-BLED score | 2.0 ± 0.9 | 2.1 ± 0.9 | 2.0 ± 1.0 | 2.0 ± 0.9 | 1.9 ± 0.9 | <0.001 |
| **AF type** |  |  |  |  |  | 0.263 |
| Paroxysmal | 470 (41.3) | 133 (39.2) | 321 (42.2) | 31 (41.9) | 13,116 (42.1) |  |
| Persistent | 341 (29.9) | 107 (31.6) | 221 (29.1) | 25 (33.8) | 8647 (27.8) |  |
| Long-standing persistent/permanent | 328 (28.8) | 99 (29.2) | 218 (28.7) | 18 (24.3) | 9373 (30.1) |  |
| **Comorbidities** |  |  |  |  |  |  |
| Cerebrovascular disease | 296 (26.0) | 93 (27.4) | 190 (25.0) | 23 (31.1) | 7007 (22.5) | 0.006 |
| Thromboembolism-related diseases | 141 (12.4) | 47 (13.9) | 90 (11.8) | 10 (13.5) | 2644 (8.5) | <0.001 |
| Active cancer | 161 (14.1) | 54 (15.9) | 98 (12.9) | 13 (17.6) | 3408 (10.9) | <0.001 |
| Dementia | 97 (8.5) | 31 (9.1) | 58 (7.6) | 12 (16.2) | 2415 (7.8) | 0.347 |
| Fall within 1 year | 102 (9.0) | 33 (9.7) | 65 (8.6) | 5 (6.8) | 2245 (7.2) | 0.011 |

Data are n (%) or mean ± standard deviation. ^†^Comparison of groups with (inclusive of patients with upper GI bleeding, lower GI bleeding, and unspecified site bleeding) and without GI bleeding.

Abbreviations: AF, atrial fibrillation; CHADS_2,_ congestive heart failure, hypertension, age ≥75, diabetes, stroke (doubled); CHA_2_DS_2_-VASc, congestive heart failure, hypertension, age ≥75 (doubled), diabetes, stroke (doubled), vascular disease, age 65 to 74 and sex category (female); DOAC, direct oral anticoagulant; GI, gastrointestinal; HAS-BLED, hypertension, abnormal liver/renal function, stroke history, bleeding history or predisposition, labile international normalised ratio, elderly, drug/alcohol usage; PPI, proton pump inhibitor.

**Supplementary Table S2.** Multivariate analysis using the Cox proportional hazards model for gastrointestinal bleeding events by site, excluding patients with off-label doses of DOAC.

| **Factors^†^** | **Overall GI bleeding** | | **Upper GI bleeding** | | **Lower GI bleeding** | |
| --- | --- | --- | --- | --- | --- | --- |
|  | **HR (95% CI)** | ***p*-value** | **HR (95% CI)** | ***p*-value** | **HR (95% CI)** | ***p*-value** |
| No-OACs | 0.65 (0.49, 0.86) | 0.003 | 0.61 (0.36, 1.03) | 0.066 | 0.69 (0.49, 0.98) | 0.039 |
| Use of DOAC (excluding off-label doses) | 1.00 (0.87, 1.14) | 0.980 | 1.00 (0.77, 1.29) | 0.983 | 1.04 (0.87, 1.24) | 0.683 |

**^†^**Reference: Warfarin

Abbreviations: CI, confidence interval; DOAC, direct oral anticoagulants; GI, gastrointestinal; HR, hazard ratio; OAC, oral anticoagulant.
